# Immediate Recognition and Surgical Treatment of Iatrogenic Acute Type A Aortic Dissection Is Associated with Low Hospital Mortality and High Intermediate-Term Survival

**DOI:** 10.31083/j.rcm2304140

**Published:** 2022-04-12

**Authors:** Yulin Wang, Fangyu Liu, Kai Song, Hao Lai, Yongxin Sun, Jun Li, Chunsheng Wang, Qiang Ji

**Affiliations:** ^1^Department of Cardiovascular Surgery, Zhongshan Hospital Fudan University, 200032 Shanghai, China; ^2^Shanghai Municipal Institute for Cardiovascular Diseases, 200032 Shanghai, China

**Keywords:** iatrogenic, acute type A aortic dissection, cardiac surgery

## Abstract

**Background:**

To investigate short- and intermediate-term outcomes of immediate (on table) recognition and surgical treatment of iatrogenic acute type A aortic dissection (ATAD) that occurred during the course of the cardiac surgical procedures.

**Methods:**

Of 23,143 adult patients undergoing cardiac surgical procedures at our institution from January 2016 to December 2020, 21 (0.09%) suffered from intraoperative iatrogenic ATAD and underwent immediate aortic repair. Their clinical characteristics, in-hospital outcomes and follow-up results were analyzed.

**Results:**

Among the 21 patients, 13 (61.9%) suffered from hypertension, and 14 (66.7%) had a dilated ascending aorta. In-hospital mortality was 9.5%, and new onset of permanent neurologic deficit was recorded in one patient. During a median follow-up of 36.0 months, all 18 follow-up patients survived without repeated surgeries. A follow-up computed tomography (CT) examination revealed a residual false lumen in the aortic arch in 3 patients and in the descending aorta in 8, with residual false lumen perfusion in one.

**Conclusions:**

Immediate recognition and surgical repair of ATAD that developed as a complication during cardiac surgical procedures are associated with low mortality and high intermediate-term survival.

## 1. Introduction

Acute type A aortic dissection (ATAD) is a life-threatening medical emergency with an incidence in western countries between 50–70/1000,000 residents [1]. Iatrogenic ATAD (iATAD) is a relatively rare but life-threatening complication associated with cardiac surgery. It usually occurs during all types of cardiac surgery (hereinafter as the “intraoperative iATAD”), with the frequencies of occurrence of 0.06–0.23% [2, 3]. Intraoperative iATAD is associated with a high mortality, which is reported to reach 48% [4]. The high mortality could be attributed to the difficulties in the immediate and accurate diagnosis of iATAD, the difficulties in timely adjustment on the alternative cardiopulmonary bypass (CPB) access, unplanned cerebral and myocardial protection, and so on [4]. The mortality associated with this lethal complication decreases along with raised awareness and more experience on this condition; nevertheless, it is still nearly twice over that reported for spontaneous aortic dissection [2, 5, 6, 7]. Accordingly, it is necessary to emphasize the importance of prompt diagnosis to avoid delay on this fatal condition and immediate surgical repair. We hypothesize that immediate recognition and surgical repair of intraoperative iATAD are linked with low hospital mortality and high intermediate-term survival. Through analyzing the data of consecutive, documented patients who presented with intraoperative iATAD and underwent immediate surgical aortic repair in our institution between 2016 and 2020, we aims to investigate short- and intermediate-term outcomes of immediate (on table) diagnosis and surgical treatment of iATAD that occurred during the course of the cardiac surgical procedure. A small number of previous reports have attached importance to intraoperative iATAD but have been limited by a small sample size, a long time span, and a high perioperative mortality, making this 5-year single-center experience valuable to describe the clinical characteristics of this challenging patient group and to evaluate the early results.

## 2. Materials and Methods

### 2.1 Patient Characteristics

Between January 2016 and December 2020, consecutive patients aged ≥18 years who underwent surgical repair for ATAD in this center were reviewed. Eligibility criteria for surgery included: (1) presenting with intraoperative iATAD; and (2) receiving immediate aortic repair. In this study, intraoperative iATAD was defined as an iATAD occurring due to a cardiac surgical procedure and during the course of the cardiac surgical procedure. Intraoperative iATAD was diagnosed via intraoperative vision and by using transesophageal echocardiographic (TEE) assessment of the aorta. Exclusion criteria included dissection occurring after cardiac surgery, dissection appearing after cardiac catheterization procedures, and iATAD associated with procedures other than cardiac surgery. This study did not include patients with spontaneous type A aortic dissection.

### 2.2 Surgical Procedures

The surgical objectives for intraoperative iATAD repair were resection of the primary aortic entry tear with the dissected aorta as much as possible and restoration of flow into the true lumen. Rapid suspension of CPB was performed as soon as the diagnosis was confirmed. The true lumen within the aortic arch was the preferred option of the location of arterial recannulation. We could perform this procedure by direct vision into the aorta or by repeated needle puncture guided by TEE. Then the guidewire followed by a cannula was guided into the true lumen. It may be difficult to differentiate the true from the false lumen, especially when the dissection involved the entire aorta. A femoral artery, alternatively, was utilized to re-establish CPB using a *Seldinger* technique. Femoral cannulation was preferred over subclavian or other peripheral arteries, as it was easy and fast to expose with enough workspace available for the second surgeon [8].

After completion of recannulation, CPB was restarted and deep hypothermia technique was applied in order to assess the extent of the dissection and repair it with individualized operation methods during a short circulatory arrest time while the aorta was exposed. Before the circulatory arrest, core body temperature was controlled to 18–25 ^∘^C rectally, varying from different surgeons. INVOS 5100C monitor (Somanatics Corporation, Troy, MI, USA) was used to real-time assess the cerebral oxygenation during the cooling stage. According to the left cerebral oxygenation, right antegrade cerebral perfusion via direct cannulation of the brachiocephalic trunk or bilateral antegrade cerebral perfusion via direct cannulation of both the brachiocephalic trunk and the left common carotid artery was conducted at a flow rate of 10 mL/kg/min.

TEE evaluation and the operative findings were valued on the determination of the extent of aortic repair. Arch reconstruction was performed in patients whose dissected intima extended into or beyond the arch. When there is a primary entry or secondary re-entry tear in the aortic arch, total arch reconstruction was arranged. Partial arch reconstruction was reserved for the rest. Proximal aortic root procedures were carried out during the rewarming stage in patients receiving arch reconstruction. The ascending aorta was replaced by a vascular prosthesis with sutures in all patients. Our technique for reinforcing the distal and proximal anastomoses was to place individual pledgetted mattress sutures along the circumference of the anastomosis over the first suture line. When there was a primary entry or secondary re-entry tear in the aortic sinus wall, aortic root repair or replacement may be required. In case of coronary malperfusion, coronary artery bypass grafting may be considered. In fact, coronary malperfusion was difficult to diagnose intraoperatively. When the dissection membrane extended into coronary ostium, coronary malperfusion should be suspected when any of the following were observed: localized wall motion abnormalities on transesophageal echocardiography; and the presence of an epicardial hematoma adjacent to the coronary ostium.

### 2.3 Study Outcomes

In-hospital outcomes included mortality, new-onset stroke, low cardiac output syndrome, prolonged mechanical ventilation, acute kidney injury requiring hemodialysis, sepsis, reoperation for bleeding, and gastrointestinal complications. In-hospital mortality was defined as any death during the same hospitalization or within 30 days after the procedure [9]. New-onset stroke was defined as any new focal or global, temporary or permanent neurological deficit with new radiologic findings [10]. Low cardiac output syndrome was defined as the requirement for mechanical circulatory support and/or inotropic support for inability to discontinue CPB or for longer than 30 min after the patient was returned to intensive care unit to maintain the systolic blood pressure >90 mmHg and the cardiac index >2.2 L/min/$m^{2}$ [11]. Prolonged mechanical ventilation was defined as the duration of mechanical ventilation of more than 48 hours or reintubation following cardiac surgery [10]. In addition, length of intensive care unit (ICU) stay and length of postoperative hospital stay were also recorded.

Follow-up data included intermediate-term survival, reoperation, New York Heart Association (NYHA) functional class, and the appearance of the residual aortic false lumen with or without perfusion. Intermediate-term survival was defined as survival at 3 years after surgical repair of intraoperative iATAD. The appearance of the residual aortic false lumen with or without perfusion was determined by using a computed tomographic (CT) scan.

### 2.4 Statistical Analysis

This is a single-center, retrospective-prospective observational study. The study protocol was approved and supervised by the Ethics Committee of Zhongshan Hospital Fudan University (No. B2021-274R). All the procedures during the study were consistent with the Declaration of Helsinki. Baseline characteristics of included patients, features of dissection (time of discovery, localization of tear, and extent of dissection), operative data (initial cardiac surgical procedures, extent of aortic surgery, aortic cross-clamp time, CPB time, and cerebral protection strategy), and in-hospital outcomes were retrieved from our electronic medical records database and were registered using a standard case reporting form. Patients were routinely followed up at three and six months after surgery, as well as at 6-month intervals after that. Follow-up data were gathered by clinic visits, online feedbacks, short message service or telephone interviews. The datasets were then double-checked for plausibility by an independent database-monitoring center. Only completed and verified datasets were used for statistical analysis.

Statistical analysis was performed with SPSS statistical software version 22.0 (SPSS Inc., Chicago, IL, USA). Categorical variables were expressed as frequency distributions and single percentages. The normality distribution of the variables was determined by using the quantile-quantile (QQ) plot. Normally distributed continuous variables were expressed as the mean and standard deviation and non-normally distributed continuous variables were expressed as median and inter-quartile range (IQR). The cumulative survival curve was conducted by the Kaplan–Meier method.

## 3. Results

### 3.1 Study Population

**Fig. 1.**
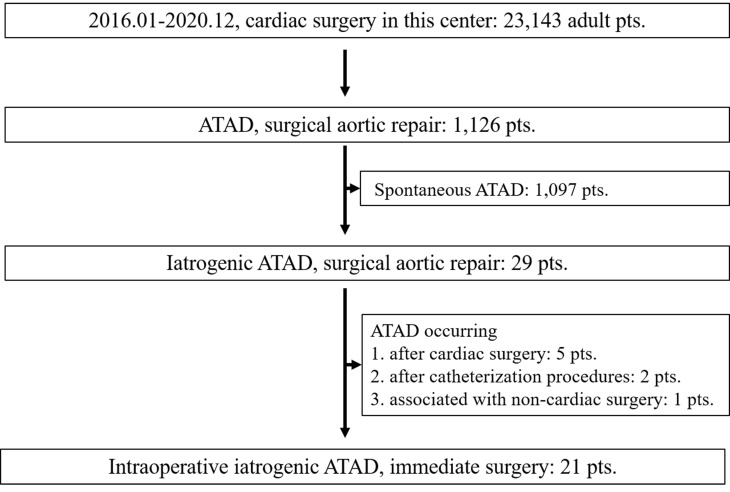
**Flow chart for the selection of study population**. 21 of 23,143 patients who underwent cardiac surgical procedures in our institution during the study period were selected for intraoperative iatrogenic ATAD study. pts., patients; ATAD, acute type A aortic dissection.

A total of 23,143 adult patients underwent cardiac surgical procedures in our institution during the study period. As presented in Fig. 1, 21 (0.09%) eligible patients were finally analyzed. The baseline characteristics of this series are shown in Table 1 (Ref. [12]). There were slightly more male (52.4%) than female patients (47.6%) with a median age of 65.0 (IQR, 56.5–73.0) years. No instance of chronic kidney disease and/or hemodialysis before surgery was recorded. It was noteworthy that dilated ascending aorta (the maximum diameter of ascending aorta of more than 40 mm measured by using transthoracic echocardiography or CT imaging) was observed in 14 (66.7%) patients with intraoperative iATAD.

**Table 1.** **Baseline characteristics**.

Variables			Frequencies (%) or mean (SD)/median (IQR)
Number of patients			21
Age (years), median (IQR)			65.0 (56.5–73.0)
Gender (Males/Females)			11/10
Obesity (Body mass index >30 kg/$m^{2}$)			2 (9.5%)
Recent smoking			1 (4.8%)
Concomitant diseases			
	Hypertension		13 (61.9%)
	Diabetes mellitus		2 (9.5%)
	Coronary artery disease		2 (9.5%)
	Dyslipidemia		4 (19.0%)
	Chronic obstructive pulmonary disease		1 (4.8%)
	Peripheral vascular disease		1 (4.8%)
eGFR (mL/min/$m^{2}$), mean (SD)			77.2 (21.8)
Preoperative cardiac status			
	Atrial fibrillation		5 (23.8%)
	Previous cardiac surgery		1 (4.8%)
	NYHA functional class		
		II	3 (14.3%)
		III	15 (71.4%)
		IV	3 (14.3%)
	LVEF (%), mean (SD)		61.1 (7.2)
	LVEF <50%		3 (14.3%)
	LVEDD (mm), mean (SD)		57.1 (5.4)
	Dilated ascending aorta (>40 mm)		14 (66.7%)
	Pulmonary hypertension		4 (19.0%)
Additive EuroSCORE, median (IQR)			4.0 (2.0–6.0)
Logistic EuroSCORE (mortality %), median (IQR)			3.0 (1.8–5.3)
Indication for initial cardiac surgery			
	Congenital bicuspid aortic valve		7 (33.3%)
	Degenerative heart valve disease		3 (14.3%)
	Rheumatic heart valve disease		2 (9.5%)
	Infective endocarditis		2 (9.5%)
	Marfan’s syndrome		2 (9.5%)
	Takayasu arteritis		2 (9.5%)
	Coronary artery disease		1 (4.8%)
	Aorta true aneurysm		1 (4.8%)
	Hypertrophic obstructive cardiomyopathy		1 (4.8%)
Initial cardiac surgical procedures			
	AVR		7 (33.3%)
	MV surgery		6 (28.5%)
	AVR with MV surgery		3 (14.3%)
	Bentall’s procedure		3 (14.3%)
	Off-pump CABG		1 (4.8%)
	TAR (for aortic arch true aneurysm)		1 (4.8%)
Time of occurrence			
	Before onset of CPB		5 (23.8%)
	During CPB		8 (38.1%)
	After the discontinuation of CPB		8 (38.1%)
Localization of entry tear			
	Aortic annulation		10 (47.5%)
	Cardioplegia cannulation		8 (38.1%)
	Axillary annulation		1 (4.8%)
	Anastomosis of saphenous vein to aorta		1 (4.8%)
	Aortotomy		1 (4.8%)
Extent of aortic dissection involvement of			
	Ascending aorta		21 (100.0%)
	Aortic arch		14 (66.7%)
	Supra-aortic vessels		4 (19.0%)
	Beyond proximal thoracic aorta		8 (38.1%)

IQR, inter-quartile range; eGFR, estimated glomerular filtration rate; SD, standard deviation; NYHA, New York Heart Association (classification); LVEF, left ventricular ejection fraction; LVEDD, left ventricular endo-diastolic diameter; EuroSCORE, European system for cardiac operative risk evaluation [12]; AVR, aortic valve replacement; MV, mitral valve; CABG, coronary artery bypass grafting; TAR, total arch reconstruction; CPB, cardiopulmonary bypass.

### 3.2 Description of Dissection

All 21 patients received elective initial cardiac surgical procedures. No patients were in critical preoperative state before initial cardiac surgical procedures. One patient with previous cardiac procedure (mechanical prosthesis replacement for both mitral and aortic valves) who was diagnosed with perivalvular leakage of mitral valve underwent a repeated mitral valve surgery and developed intraoperative iATAD. Intraoperative iATAD occurred before the onset of CPB in 5 (23.8%) patients, during CPB in 8 (38.1%) patients, and after the discontinuation of CPB in 8 (38.1%) patients. The most frequent origin of the dissection was aortic cannula itself (47.5%, n = 10), followed by cardioplegia cannulation (38.1%, n = 8). The dissected intima extended proximally into the ascending aorta in all patients; and it extended distally into the aortic arch in 14 (66.7%) patients, into the supra-aortic vessels in 4 (19.0%), and beyond proximal thoracic aorta in 8 (38.1%). The features of dissection are listed in Table 1.

### 3.3 Surgical Data

Regarding proximal injury, an ascending aorta replacement was performed in all patients, and a CABG (aorta-vein graft-right main coronary artery) was conducted in one patient with coronary malperfusion resulted from the dissection extending into coronary ostium and the right main coronary artery. No aortic root repair or replacement was recorded. Regarding distal aortic repair, a partial arch reconstruction was performed in 10 (47.6%) patients and a total aortic arch reconstruction in 4 (19.0%).

The cumulative aortic cross-clamping time and cumulative CPB time was 105.5 (IQR, 94.3–140.0) minutes and 217.5 (IQR, 156.0–257.0) minutes, respectively. Circulatory arrest was performed in 14 (66.7%) patients with a mean duration of 16.8 minutes. The mean lowest rectal temperature was 22.6 centigrade. Among 14 patients undergoing circulatory arrest, 13 received antegrade cerebral perfusion (right in 11 patients and bilateral in 2 patients). One patient with ascending aorta replacement plus partial arch reconstruction, who received a circulatory arrest time of 8 minutes and a lowest rectal temperature of 19.9 centigrade, did not receive antegrade or retrograde cerebral perfusion. Surgical data of this case series are summarized in Table 2.

**Table 2.** **Aortic dissection operative data**.

Variables		Frequencies (%) or mean (SD)/median (IQR)
Extent of aortic surgery		
	Isolated ascending aorta replacement	7
	Ascending aorta + partial arch replacement	10
	Ascending aorta replacement + TAR	3
	Ascending aorta replacement + TAR + CABG	1
Cumulative CPB (min), median (IQR)		217.5 (156.0–257.0)
Cumulative ACC (min), median (IQR)		105.5 (94.3–140.0)
Circulatory arrest		
	Number of patients	14
	Duration (min), mean (SD)	16.8 (3.6)
Lowest temperature (rectal)		
	Number of patients	14
	Temperature (centigrade), mean (SD)	22.6 (1.2)
Cerebral perfusion		
	Antegrade	13
	Right	11
	Bilateral	2

SD, standard deviation; IQR, inter-quartile range; TAR, total arch reconstruction; CABG, coronary artery bypass grafting; CPB, cardiopulmonary bypass; ACC, aortic cross-clamping.

### 3.4 In-Hospital Outcomes

Two (9.5%) patients died of the rupture of aortic root on the first day following surgery and of the rupture of abdominal aortic dissection on the fifth day after surgery, respectively. New-onset stroke was observed in 4 (19.0%) patients, including 3 with temporary neurological deficit lasting less than 3 weeks and one (4.8%) with neurologic deficit that persisted at the time of hospital discharge. As shown in Table 3, other perioperative complications included low cardiac output syndrome in 2 (9.5%) patients, prolonged ventilation and/or re-intubation in 9 (42.9%), acute kidney injury requiring hemodialysis in 5 (23.8%), and sepsis in 3 (14.3%). Fifteen (71.4%) patients received blood transfusion. The median ICU stay was 4.0 (IQR, 2.0–15.5) days. Nineteen (90.5%) patients recovered and were discharged, with a median length of postoperative hospital stay of 11.0 (IQR, 7.5–31.0) days.

**Table 3.** **Postoperative outcomes**.

Variables			Frequencies (%) or median (IQR)
In-hospital			n = 21
	Death		2 (9.5%)
	New-onset stroke		4 (19.0%)
		Temporary	3
		Permanent	1
	Low cardiac output syndrome		2 (9.5%)
	Blood transfusion		15 (71.4%)
	Prolonged ventilation (including re-intubation)		9 (42.9%)
	Acute kidney injury requiring hemodialysis		5 (23.8%)
	Sepsis		3 (14.3%)
	Reoperation for bleeding		1 (4.8%)
	Gastrointestinal complication		1 (4.8%)
	Length of ICU stay (d), median (IQR)		4.0 (2.0–15.5)
	Length of postop. hospital stay (d), median (IQR)		11.0 (7.5–31.0)
Follow-up			
	Number of patients		18
	Follow-up time (m), median (IQR)		36.0 (16.5–45.0)
	Survival		18 (100%)
	NYHA class		
		I	7 (38.9%)
		II	10 (55.6%)
		III	1 (5.5%)
	Residual false lumen by CT scan		
		Ascending aorta	0
		Aortic arch	3 (18.8%)
		Descending aorta	8 (50.0%)
		Abdominal aorta	4 (25.0%)
	Residual false lumen perfusion by CT scan		1 (6.3%)

IQR, inter-quartile range; ICU, intensive care unit; NYHA, New York Heart Association (classification); postop., postoperative; CT, computed tomography.

### 3.5 Follow-Up Results

**Fig. 2.**
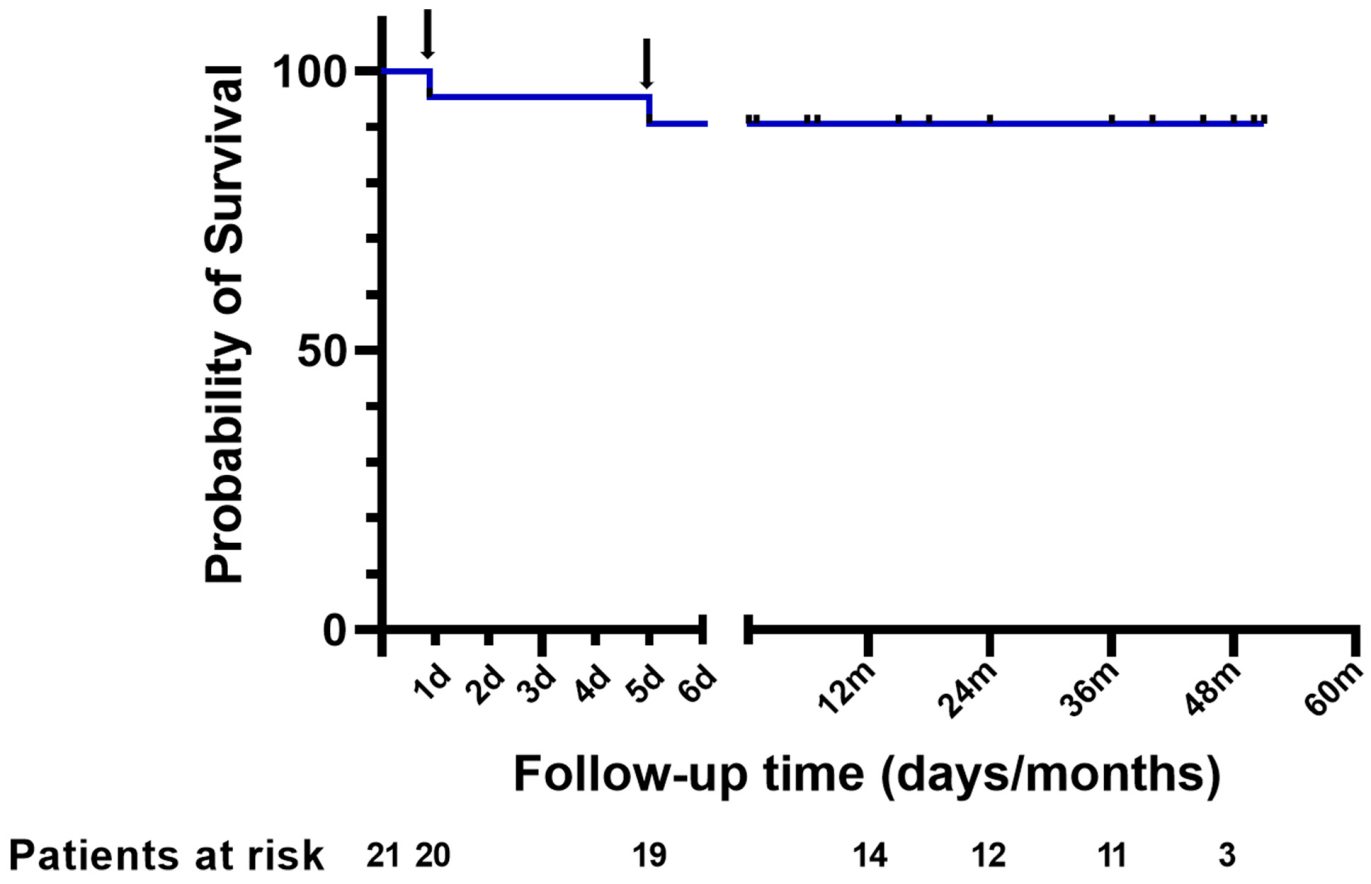
**Cumulative survival after surgery**. One patient died of the rupture of aortic root on the first day after surgery and one patient died of the rupture of abdominal aortic dissection on the fifth day after surgery (black arrow).

A total of 18 patients, accounting for 94.7% of discharged patients, received a follow-up visit. As presented in Fig. 2, Kaplan–Meier curves revealed that all 18 follow-up patients survived during the median duration of 36.0 (IQR, 16.5–45.0) months. No repeated surgery was recorded. Seventeen out of 18 follow-up patients were classified as NYHA class I–II. A follow-up CT examination at 3–6 months after discharge was available in 16 patients (84.2% of survivors) and showed a residual false lumen in the aortic arch in 3 (18.8%) patients, in the descending aorta in 8 (50.0%), and in the abdominal aorta in 4 (25.0%). The residual false lumen perfusion (the descending aorta and the abdominal aorta) was recorded in one (6.3%) patient, but the maximum diameter of the aorta was less than 40 mm. We classified the patient as NYHA class II and proceeded with dynamic evaluation and CT examination. No residual false lumen was found in the ascending aorta.

## 4. Discussion

This study has demonstrated that immediate (on table) diagnosis and surgical repair of ATAD that occurred during the course of the cardiac surgical procedures are associated with low hospital mortality and high intermediate-term survival. The most devastating outcome of surgical treatment for intraoperative iATAD was early mortality. The published small case series have found that surgical therapy for iATAD was related to a higher perioperative mortality, ranging from 20 to 48%, than surgery for spontaneous aortic dissection [4, 13, 14]. In contrast to the 20–48% mortality reported in previous studies, we achieved an encouraging result with an in-hospital mortality of only 9.5%. Our favorable result may be attributed to factors as follows. The first factor was an experienced cardiac surgical team consisting of surgeons, anesthesiologists, and perfusionists, in dealing with aortic dissection. In our center, open surgical repair of spontaneous type A aortic dissection has become a common practice in these years with an average procedure volume of over 200 cases each year. The second factor was prompt diagnosis. During cardiac surgical procedures, potential onset of iATAD originated in the ascending aorta should be alerted when the following signs were observed: blue discolored aorta caused by intramural hematoma and active bleeding from the incisions or needle puncture or cannulation sites in the aorta; monitored blood pressure changed unexpectedly in a short period (usually decreased or altered waveform) in systemic arterial lines such as radial artery; CPB-associated changes including increased circuit arterial-line pressure, excessive loss of perfusate, sudden decrease in venous return and any evidence of insufficient organ perfusion (e.g., cerebral oximetry decline, dilated pupils, electrocardiographic signs of myocardial ischemia). Although the diagnosis of all 21 patients was made during operation, no exact time elapsed from the onset of intraoperative iATAD to the diagnosis was recorded. In our experience, the diagnosis should be made within minutes. All cardiac surgeons in this center were on high alert against the occurrence of this rare but life-threatening complication of cardiac surgical procedures. If there was any doubt as to whether intraoperative iATAD occurred, TEE assessment of the aorta should be performed immediately. TEE evaluation was of great value for prompt diagnosis and was frequently used to assist surgical management. The third factor was prompt and appropriate management. Management varied depending on the origin and the extent of the dissection. Timely alternative access for CPB, prompt suspension of perfusion into the false lumen and resumption of perfusion into the true lumen, application of deep hypothermia, and the addition of cerebral protection strategies, were key components of management. Notably, both two patients died of dissection rupture instead of cardiogenic shock, which is a main cause of death reported in the literature [15], suggesting that aortic root repair should be paid more attention to and early postoperative blood pressure control should be emphasized. The fourth factor was short cumulative aortic cross-clamping time. In a retrospective observational study of 363 patients who underwent ATAD repair, Beliaev and Bergin [16] have found that the duration of cardiac ischemia in ATAD repair was linked to operative mortality. They reported that cardiac ischemic time of less than 150 minutes may be associated with lower in-hospital mortality and better long-term survival. In this case series, the median cumulative aortic cross-clamping time was only 105.5 minutes, which was shorter than previously published results [17, 18]. So, short cumulative aortic cross-clamping time may contribute to achieving the favorable results. In addition, no death or repeated surgery was recorded during a follow-up of 36 months, suggesting that immediate surgical repair of intraoperative iATAD achieved a favorable intermediate-term outcome.

Coronary malperfusion was reported to present in 27.4% of all iATAD patients, but it occurred more frequently in patients with cardiac catheterization-induced iATAD [6]. In this series, coronary malperfusion was observed in only one (4.8%) patient. The low rate may be due to the fact that this study included only patients suffering from iATAD during cardiac surgery, with no patients with cardiac catheterization-induced iATAD.

Hypoxic brain injury was a catastrophic outcome following aortic repair [4, 19]. The key to avoiding this adverse outcome included early diagnosis, prompt attention to maintenance of cerebral perfusion and protection, and minimizing the duration of circulatory arrest. Although four (19.0%) patients in this series suffered from new onset stroke, only one (4.8%) developed neurologic deficit that persisted beyond hospital discharge. We believed that early recognition of intraoperative iATAD, rapid resumption of CPB, prompt induction of deep hypothermia, short circulatory arrest time (a mean of 16.8 minutes in this series), and the addition of selective antegrade cerebral perfusion may contribute to the reduction of the risk of hypoxic brain injury.

In this series, the relatively high rate of residual false lumen following repair suggested patients with intraoperative iATAD required lifelong clinical follow-up [6], although the incidence of residual false lumen perfusion was low and the risk of late death appeared low.

Intraoperative iATAD was a rare complication, with a reported occurrence of 0.04–0.23% [4, 8, 20, 21, 22, 23]. In this case series, the incidence of intraoperative iATAD was 0.09%, consistent with previous reports. The occurrence of iATAD varied significantly depending on the site of the arterial cannulation [2]. In this case series, aortic cannula itself and cardioplegia cannulation were most frequent original sites of dissection, accounting for 85.7%, consistent with a previous meta-analysis [24].

Singh and Mehta [25] provided a list of potential risk factors associated with increased incidence of intraoperative iATAD. In this case series, dilated ascending aorta was observed in 66.7% of patients. According to our intraoperative observation and experience, dilated ascending aorta may be associated with a vulnerable ascending aorta. The median age of this series was 65.0 years. Potential explanations for the higher age of patients with intraoperative iATAD may be the increasing fragility of aortic walls in the elderly patients [7]. Theoretically, any trauma of a vulnerable aorta during surgical procedures (e.g., clamping, cannulation, incisions, anastomosis) could lead to injury the intact intima, with pulsatile blood flow flushing inside the lumen, which perpetuated the dissection [26, 27]. However, further studies were required.

The principal limitation of this study was its retrospective design with a small sample size. However, the rarity of this dangerous iatrogenic disease made such a study design necessary. Our center performed a high volume of adult cardiac surgical procedures, which enabled us to collect data on a relatively large study population of surgically treated intraoperative iATAD. Another limitation was the lack of identification of independent predictors of intraoperative iATAD, because of the rarity of its occurrence (<0.1% of patients) and the small sample size (n = 21), and because of the multitude of different cardiovascular interventions during which it occurred.

## 5. Conclusions

Immediate (on table) recognition and surgical repair of ATAD that developed as a complication during cardiac surgical operations are associated with low mortality and high intermediate-term survival. Further comparative studies are required to confirm our study results.
